# Activation of the p11/SMARCA3/Neurensin-2 pathway in parvalbumin interneurons mediates the response to chronic antidepressants

**DOI:** 10.1038/s41380-021-01059-4

**Published:** 2021-03-15

**Authors:** Gali Umschweif, Lucian Medrihan, Kathryn A. McCabe, Yotam Sagi, Paul Greengard

**Affiliations:** Laboratory of Molecular and Cellular Neuroscience, The Rockefeller University, New York, NY USA

**Keywords:** Neuroscience, Depression

## Abstract

The delayed behavioral response to chronic antidepressants depends on dynamic changes in the hippocampus. It was suggested that the antidepressant protein p11 and the chromatin remodeling factor SMARCA3 mediate this delayed response by inducing transcriptional changes in hippocampal neurons. However, what target genes are regulated by the p11/SMARCA3 complex to mediate the behavioral response to antidepressants, and what cell type mediates these molecular changes remain unknown. Here we report that the p11/SMARCA3 complex represses Neurensin-2 transcription in hippocampal parvalbumin-expressing interneurons after chronic treatment with Selective Serotonin Reuptake Inhibitors (SSRI). The behavioral response to antidepressants requires upregulation of p11, accumulation of SMARCA3 in the cell nucleus, and a consequent repression of Neurensin-2 transcription in these interneurons. We elucidate a functional role for p11/SMARCA3/Neurensin-2 pathway in regulating AMPA-receptor signaling in parvalbumin-expressing interneurons, a function that is enhanced by chronic treatment with SSRIs. These results link SSRIs to dynamic glutamatergic changes and implicate p11/SMARCA3/Neurensin-2 pathway in the development of more specific and efficient therapeutic strategies for neuropsychiatric disorders.

## Introduction

Treatment with selective serotonin reuptake inhibitors (SSRIs) is the first-line therapy for Major Depressive Disorder (MDD), and is commonly used for other neuropsychiatric disorders, including anxiety, obsessive compulsive, eating, and post-traumatic stress disorders [1]. A typical delayed clinical response to SSRIs, ranging from 2 to 6 weeks [2], indicates that the mechanism of action of these agents requires slow adaptive changes, including neuronal plasticity. Indeed, SSRIs-induced neuroplasticity was extensively documented in the limbic system, predominantly in the dentate gyrus (DG) of the hippocampus [3]. While many studies highlight the effect of SSRIs on the proliferation of newborn granule neurons [4], functional changes in mature granule cells seem to play a major role in mediating the response to SSRIs [5–7]. Chronic SSRI treatment induces enhancement in the synaptic strength of projections from the entorhinal cortex onto mature granule cells [6, 8–10]. Moreover, chronic treatment with SSRI also reverses the maturation state of granule cells. This effect is characterized by enhanced expression of markers of premature neurons and increased neuronal activity typical to immature granule cells [11]. Similar granule cell de-maturation response was observed following electroconvulsive procedure [12], supporting its role in the action of antidepressants.

The molecular mechanisms underlying these plastic changes in the DG by SSRIs remain mostly unknown. The neurotropic hypothesis suggests an important role for brain-derived neurotrophic factor (BDNF) in the hippocampus and in the prefrontal cortex [13]. In the hippocampus, BDNF expression is enhanced following chronic treatment with antidepressants, especially in the DG [14–16]. Upon its upregulation, BDNF activates downstream kinases and transcriptional factors, which correlates with the delayed beneficial effect of SSRIs [17, 18]. Moreover, downregulation of BDNF gene expression in the DG blocked the behavioral response to chronic antidepressants [15], suggesting that BDNF expression in the DG is crucial for the efficacy of the treatment. We previously reported that chronic SSRIs and the subsequential induction in BDNF result in the upregulation of the antidepressant protein p11 [19, 20]. p11 expression in the hippocampus is highly linked toresilience to stress and to the therapeutic effects of SSRIs [20–23]. Following chronic SSRIs, p11 binds SWI/SNF-related, matrix-associated, actin-dependent regulator of chromatin 3 (SMARCA3) to mediate the behavioral response to the treatment [21, 24]. It was suggested that by binding p11, SMARCA3 could modulate gene transcription to evoke plastic changes in the hippocampal DG cells [24]. However, the target genes of p11-SMARCA3 complex, as well as their involvement in neuronal plasticity and behavior, remain unknown. We recently reported that in the DG, SMARCA3 induces the repression of Neurensin-2 [25]. This protein regulates both depression-related behavior and functional plasticity of the interneurons it is expressed in. However, whether Neurensin-2 expression is regulated by chronic antidepressants remains an open question.

## Materials and methods

### Animals

All procedures were approved by The Rockefeller University Institutional Animal Care and Use Committee and were in accordance with the National Institutes of Health Guide for the Care and Use of Laboratory Animals guidelines. Mice were maintained on a C57BL/6 background and were kept on a 12 h light/dark cycle with food and water ad libitum. C57BL/6 mice were purchased from Jackson Laboratories. For social defeat studies, CD-1 mice were purchased from Charles River Laboratories. SMARCA3 conditional KO mice were generated by crossing mice that harbor the Hltf gene flanked by lox cassettes [24] with Pvalb^tm1(cre)Arbr/J^ mice, CCK^tm1.1(cre)Zjh/J^, or with Nestin^(B6.Cg(SJL)-TN(NesCre)1Kln/J)^ mice. Previously published p11 KO mice [20] were used for western blot and Neurensin-2 KO (companion paper) mice were used for behavior. For electrophysiology experiments, Neurensin-2 KO mice were bred with Pvalb^tm1(cre)Arbr/J^ mice to generate homozygote deletion of Neurensin-2 with Cre expression in PV cells. For immunohistochemistry and for electrophysiology experiments, PV^TRAP^ mice were created by crossing Pvalb^tm1(cre)Arbr/J^ mice with mice expressing loxP-stop-loxP-EGFP-RPL10a sequence in the Eef1α1 promoter (EEF1A1–LSL.EGFPL10) [26]. CCK^tm1.1(cre)Zjh/J^ and Pvalb^tm1(cre)Arbr/J^ mice were used for viral injections. For western blot, qPCR and behavior experiments, only males were used. For immunohistochemistry and electrophysiology, both males and females were used, with no differences observed between genders.

### Drug administration

Chronic fluoxetine treatment was given to 9 weeks old C57BL/6 mice and included 21 days of free drinking of fluoxetine (0.167 mg/ml) in 1% saccharine or vehicle (1% saccharine), as previously described [22]. Using this regimen, mice receive 16–23 mg/kg/day. After restraint stress, or in stress-naïve mice, fluoxetine or vehicle was administrated by a daily intraperitoneal injection at 20 mg/kg dose, as previously described [24]. For hippocampal p11 levels analysis, vehicle or citalopram were injected intraperitoneally at dose of 10 mg/kg/day for 14 days. For examination of Nrsn2 levels after chronic treatment, Fluoxetine (20 mg/kg/day), imipramine (20 mg/kg/day), citalopram (20 mg/kg/day), or mirtazapine (10 mg/kg/day) were injected intraperitoneally for 14 days. For examination of Nrsn2 levels after acute treatment, vehicle, fluoxetine (20 mg/kg) or ketamine (10 mg/kg) were injected intraperitoneally and brains were collected 30 min after the injection.

Cell culture, TRAP samples preparation and analysis, stress models, behavioral assays, immunohistochemistry, Western blotting, AAV preparation, stereotaxic delivery and electrophysiology were performed as described in the Extended Data Supplementary Methods Section.

## Results

### Chronic antidepressants regulate Neurensin-2 levels via the p11-SMARCA3 pathway

**Fig. 1:**
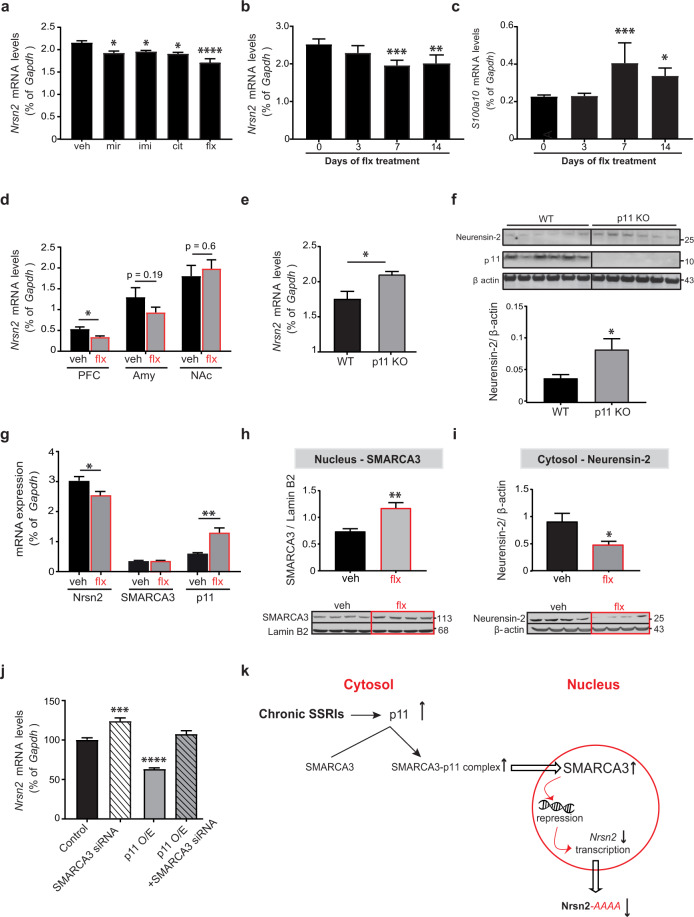
Chronic antidepressant treatment and p11 downregulate Neurensin-2. **a** Bar graph of qPCR analysis of Nrsn2 mRNA expression in the hippocampus after 14 days of daily intraperitoneal injection of vehicle (veh), mirtazapine (mir), imipramine (imi), citalopram (cit) or fluoxetine (flx). One-way ANOVA, *F* (4, 20) = 9.34, *P* = 0.0002; *p = 0.018 for mir, *p = 0.035 for imi, *p = 0.01 for cit, ****p = 0.0001 for flx. *n* = 5/group. **b**, **c** Bar graphs showing qPCR analysis of Nrsn2 (**b**) and p11 (**c**) transcripts in the hippocampus before (0) and after 3, 7, and 14 days of fluoxetine treatment (20 mg/kg/day). **b** One-way ANOVA; *F* (3, 16) = 10.67, *P* = 0.0004; **p = 0.001, ***p = 0.0004. **c** One-way ANOVA, *F* (3, 16) = 11.16, *P* = 0.0003; *p = 0.022, ***p = 0.0005. *n* = 5/group. **d** Bar graphs showing qPCR analysis of Nrsn2 mRNA in the prefrontal cortex (PFC), amygdala (Amy), and nucleus accumbens (NAc) following chronic flx (20 mg/kg/day, 14 days). Unpaired *t* test, *p = 0.017. *n* = 5/group. **e** Bar graph showing qPCR analysis of *Nrsn2* mRNA expression. Bulk RNA in the hippocampus was extracted from WT and p11 KO mice. *t* test, *p = 0.04. *n* = 5/group. **f** Western blot scan (top) and quantification (bottom) of Neurensin-2 protein expression in hippocampi from WT and p11 KO mice. *t* test, *p = 0.028, *n* = 6, 6 mice. Molecular weights are indicated in KDa. **g** Bar graph of qPCR analysis of Nrsn2, p11, and SMARCA3 mRNA expression. Bulk RNA from mice hippocampi was analyzed following 21 days of fluoxetine (flx, indicated by red line) or vehicle (veh) treatment. Unpaired *t* test, *p = 0.039, **p = 0.004, *n* = 6, 5. Top, schematic of experimental design. **h**–**i** Western blot scans (bottom) and quantification (top) of nuclear SMARCA3 (**h**) and cytosolic Neurensin-2 (**i**) protein levels in the hippocampus of stress-naive mice treated with flx or veh. Unpaired *t* test, **p = 0.004; *p = 0.016, *n* = 6 mice/group. Molecular weights are indicated in KDa. **j** Bar graph showing qPCR analysis of Nrsn2 mRNA in N2a cells. One-way ANOVA; *F* (4, 21) = 34.9, *p* < 0.0001. ***p < 0.0005 vs. control, ****p < 0.0001 vs. control. *n* = 4–6 biological replicates/group. **k** Suggested model for the regulation of Nrsn2 by p11- SMARCA3 complex in the hippocampus. Chronic SSRIs induce p11 levels in the cytosol and the formation of p11-SMARCA3 complex. This allows nuclear accumulation of SMARCA3 and inhibition of Nrsn2 transcription.

To determine if Neurensin-2 levels in the hippocampus are altered by chronic antidepressant treatment, we measured changes in the level of its transcript (*Nrsn2*). Fourteen days of daily treatment with either mirtazapine, imipramine, citalopram or fluoxetine resulted in downregulation of *Nrsn2* (Fig. 1a). Among these antidepressant drugs, the most significant reduction in *Nrsn2* levels was observed following treatment with the SSRI fluoxetine, which resulted in downregulation of *Nrsn2* by 20.5 ± 6% (Fig. 1a). As expected, the downregulation of *Nrsn2* was accompanied by an upregulation in p11 transcript levels (*S100a10*, Fig. S1a) [19, 20]. We then examined the dynamics of the regulation of *Nrsn2* levels in the hippocampus following treatment with antidepressants. Downregulation of *Nrsn2* expression was detected after 7 days of fluoxetine treatment (Fig. 1b), a timeline that was similar to that observed for the upregulation in p11 levels (Fig. 1c). We further confirmed that a single dose of either fluoxetine or the fast-acting antidepressant, ketamine did not alter *Nrsn2* levels, supporting the idea that repeated treatment is required to induce the downregulation in its transcription (Fig. S1b). We then examined the changes in Neurensin-2 levels by antidepressants in other limbic areas. *Nrsn2* level was downregulated by chronic fluoxetine in the prefrontal cortex, but not in the amygdala and nucleus accumbens (Fig. 1d), as previously shown for p11 [27–30]. The facts that Neurensin-2 and p11 expression show opposing, simultaneous dynamics by chronic antidepressant and that they both are regulated in similar brain regions support the idea that both of these two molecules play a part in the same signaling pathway.

To test a possible role for p11 in the regulation of Neurensin-2, we examined the hippocampal expression levels of Neurensin-2 in p11 KO mice. We found that *Nrsn2* transcript and Neurensin-2 protein levels were both upregulated when p11 was deleted (Fig. 1e, f), suggesting that p11 has a role in repressing Neurensin-2 transcription. It was previously shown that chronic antidepressant treatment induces the formation of p11-SMARCA3 complex that mediates the behavioral response to this treatment [24]. In line with this, deletion of SMARCA3 in the nervous system resulted in induction in hippocampal *Nrsn2* levels (Fig. S1c), supporting the idea that the p11-SMARCA3 complex may be essential for the downregulation of *Nrsn2* following antidepressants. We also found that chronic fluoxetine treatment resulted in transcriptional upregulation of p11 and downregulation of Neurensin-2, whereas the transcription of SMARCA3 was unchanged, suggesting that the formation of the protein complex p11-SMARCA3 after chronic fluoxetine treatment does not require transcriptional induction of the latter (Fig. 1g). Therefore, the upregulation of p11 by chronic antidepressants might induce the nuclear translocation of SMARCA3. Indeed, 14 days of fluoxetine treatment resulted in a 60 ± 26% increase in SMARCA3 levels in the cell nucleus fraction (Fig. 1h), and a 46 ± 16.7% decrease in Neurensin-2 protein levels in the respective hippocampal cytosolic extract (Fig. 1i). These findings suggest that the activity of SMARCA3 on repressing *Nrsn2* transcription is increased following the induction in p11 levels. To confirm this hypothesis, we utilized an in vitro system. Downregulation of SMARCA3 in N2a cells resulted in upregulation of *Nrsn2* mRNA levels (Fig. 1j). Notably, *Nrsn2* transcript was downregulated by overexpressing p11, an effect that was dependent on intact SMARCA3 expression (Fig. 1j). Taken together, these data strongly suggest that following chronic antidepressant treatment, the p11-SMARCA3 complex is recruited to the nucleus to mediate the suppression in *Nrsn2* transcription in hippocampal neurons (Fig. 1k).

### Stress-induced alteration in the SMARCA3/Neurensin-2 pathway is restored by chronic SSRIs

**Fig. 2:**
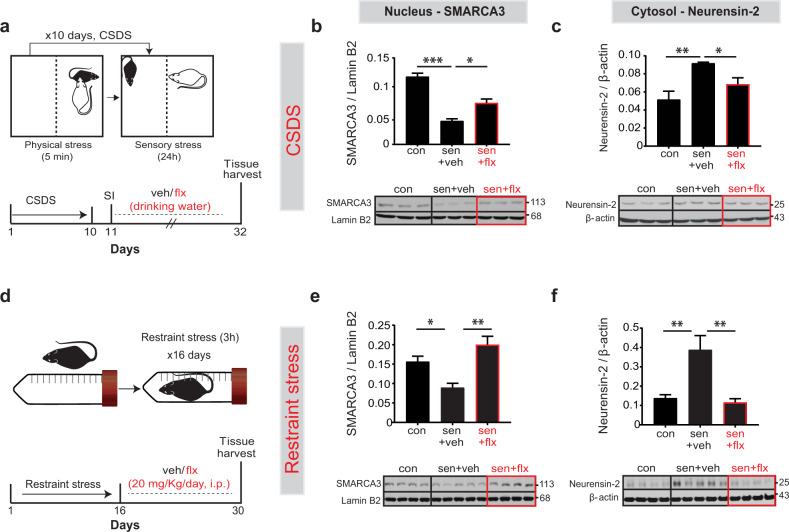
Neurensin-2 induction by stress is reversed by antidepressant treatment. **a** Schematic of the Chronic Social Defeat Stress (CSDS). Following 10 days of CSDS, stress-sensitive mice (sen) were identified using the social interaction test (SI). These mice were treated with either vehicle (veh) or fluoxetine (flx) for 21 days. **b**, **c** Western blot scans (top) and quantification (bottom) of nuclear SMARCA3 (**b**) and cytosolic Neurensin-2 (**c**) from hippocampi of non-stressed control or stress-sensitive mice. **b** Two-way ANOVA; *F* (1, 13) = 14.32. Interaction, *P* = 0.005; stress, *F* = 167.4, *P* < 0.0001; treatment, *F* = 73.92, *P* < 0.0001. *p = 0.037; ***p = 0.0009. **c** Two-way ANOVA; *F* (1, 13) = 0.06, interaction, *P* = 0.059; stress, *F* = 61.87, *P* < 0.0001; treatment, *F* = 18.65, *P* = 0.001. *p = 0.042; **p = 0.001. **d** Schematic of experimental design for restraint stress followed by 14 days of flx or veh. **e**, **f** Western blot scans (top) and quantification (bottom) of nuclear SMARCA3 (**e**) and cytosolic Neurensin-2 (**f**) from hippocampi of control, or stressed mice. **e** Two-way ANOVA; interaction, *F* (1, 13) = 0.0003, *P* = 0.95; stress, *F* = 18.74, *P* = 0.0009; treatment, *F* = 49.84, *P* < 0.0009. *p = 0.038, **p = 0.001. **f** Two-way ANOVA; interaction, *F* (1, 14) = 5.80, *P* = 0.03; stress, *F* = 11.09, *P* = 0.005; treatment, *F* = 14.78, *P* = 0.0018. **p = 0.006 for stress, **p = 0.003 for flx. *n* = 4–5 mice/group. Molecular weights are indicated in KDa.

We then tested the idea that the activation of the p11/SMARCA3/Neurensin-2 pathway might be associated with alleviating depressive-like behavior by antidepressants. To test this, we used two well-established rodent models of MDD. In the chronic social defeat stress paradigm (CSDS, Fig. 2a), we identified stress-sensitive and stress-resilient mice (Fig. S2a). Then we treated stress-sensitive mice with fluoxetine for 21 days (Fig. 2a). This treatment reduced the avoidance from a novel aggressor (Fig. S2b), increased preference to sucrose (Fig. S2c), and reduced the latency to bite a food pellet (Fig. S2d). Chronic social stress resulted in 59 ± 6.5% reduction in nuclear SMARCA3 levels, and in 78 ± 3.2% induction in cytosolic Neurensin-2 levels. Notably, chronic treatment with fluoxetine increased the accumulation of SMARCA3 in the cell nucleus of stressed mice (Fig. 2b) and fully restored the upregulation in Neurensin-2 levels (Fig. 2c). We then confirmed these molecular effects by antidepressants in the chronic restraint stress, another model of MDD (Fig. 2d). Chronic restrained stress resulted in sustained reduction in the nuclear level of SMARCA3, which was completely restored following fluoxetine treatment (Fig. 2e). At the same time, the stress led to upregulation in Neurensin-2 level in the hippocampus, whereas fluoxetine treatment resulted in full restoration of its level relative to that in stressed mice treated with the vehicle (Fig. 2f). Together, these data suggest that the p11-SMARCA3 complex represses Neurensin-2 expression following chronic SSRI treatment to alleviate the depressive state.

### p11-SMARCA3 pathway in parvalbumin-expressing interneurons mediates the response to SSRIs

**Fig. 3:**
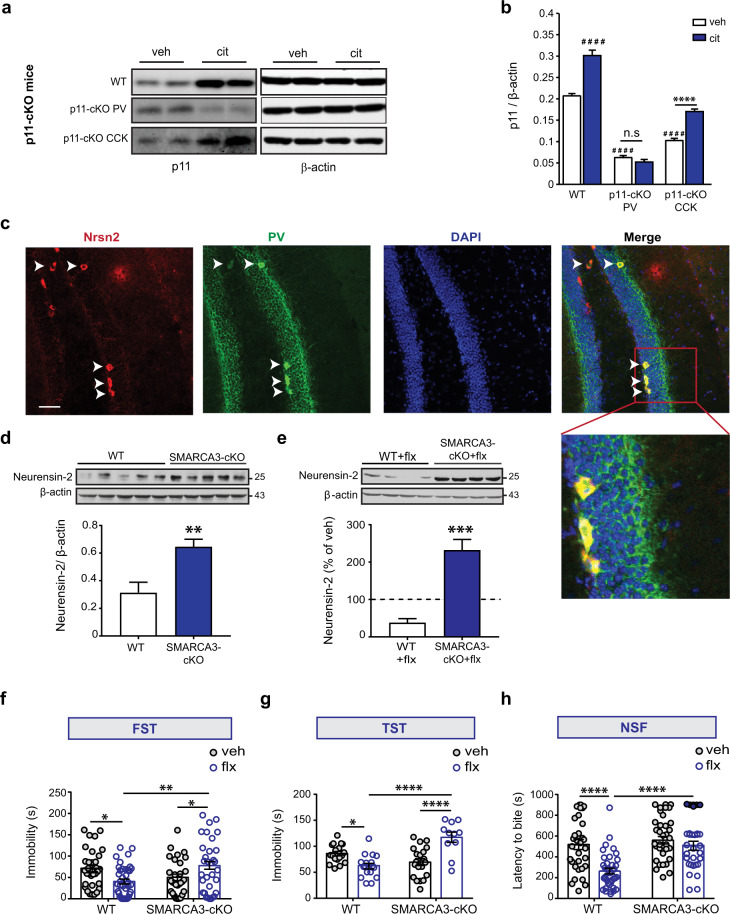
p11 and SMARCA3 in PV neurons mediate the response to chronic SSRIs. **a**, **b** Representative western blots (**a**) and bar graph summary (**b**) of p11 and β-actin levels in the hippocampus of WT mice, or in mice with conditional deletion of p11 in PV cells (p11-cKO PV) or in CCK cells (p11-cKO CCK). Mice were injected with saline (veh) or citalopram (cit) once a day for 14 days. Two-way ANOVA, interaction, *F* (2, 18) = 34.19. ****p < 0.0001. ^####^*p* < 0.0001 vs. WT treated with veh, n.s not significant. **c** Representative immunohistochemical images showing localization of Neurensin-2 protein in PV positive cells located in the DG of WT mouse. Arrowheads and box highlight immunopositive cells in the SGZ. Scale bar, 50 μm. **d** Western blot scan (top) and quantification (bottom) of hippocampal Neurensin-2 protein expression in WT mice and in mice with SMARCA3 deletion in PV cells (SMARCA3 cKO). Unpaired *t* test, **p = 0.007, *n* = 5/group. **e** Western blot scan (top) and quantification (bottom) of hippocampal Neurensin-2 protein expression in WT mice and in mice with SMARCA3 deletion in PV cells after chronic treatment with fluoxetine (flx). Dashed line indicates Neurensin-2 levels in mice treated with vehicle (100%). Unpaired *t* test, ***p = 0.0003, *n* = 4/group. **f**–**h** Behavioral tests in WT or in mice with deletion of SMARCA3 in PV cells (SMARCA3-cKO) after 21 days of treatment with vehicle (veh) or fluoxetine (flx). **f** Forced swim test (FST). Two-way ANOVA; interaction, *F* (1, 129) = 12.08, *P* = 0.0007; genotype, *F* = 0.97, *P* = 0.32; treatment, *F* = 0.5, *P* = 0.82. *p = 0.038 for WT mice, *p = 0.046 for cKO mice, **p = 0.007, *n* = 32, 39, 30, 32. **g** Tail suspension test (TST). Two-way ANOVA; *F* (1, 62) = 33.35, interaction, *P* < 0.0001; genotype, *F* = 4.19, *P* = 0.044; treatment, *F* = 8.37, *P* = 0.005. *p = 0.035, ****p < 0.0001, *n* = 18, 17, 20, 11. **h** Novelty suppressed feeding (NSF). Two-way ANOVA; interaction, *F* (1, 129) = 7.17, *P* = 0.008; genotype, *F* = 15.93, *P* = 0.0001; treatment, *F* = 14.16, *P* = 0.0003. ****p < 0.0001, *n* = 32, 39, 30, 32. Molecular weights are indicated in KDa.

The behavioral response to chronic antidepressant treatment is mediated by p11 in DG cells, including cholecystokinin (CCK) [30] and parvalbumin (PV) [23] inhibitory interneurons, and the excitatory Mossy cells [31]. Among these, the two inhibitory interneurons also express Neurensin-2 [25]. However, the cell type in which the activation of the p11/Neurensin-2 pathway by antidepressants occurs, remains unknown. To determine the cell type involved in this response to chronic antidepressants, we deleted p11 in either PV or CCK cells and measured its hippocampal upregulation in response to chronic SSRIs. The upregulation of p11 after 14 days of citalopram treatment was abolished when p11 was deleted from PV cells, but not from CCK cells (Fig. 3a, b). Using immunohistochemistry, we confirmed that the downstream target of p11/SMARCA3 pathway, Neurensin-2, is highly and selectively expressed in PV cells in the DG (Fig. 3c). To assess its role in regulating Neurensin-2 expression in PV interneurons, we deleted SMARCA3 from PV cells (SMARCA3 cKO) and validated the downregulation of SMARCA3 in these cells (Fig. S3a, b). In hippocampal lysates from SMARCA3 cKO mice, we detected an upregulation of Neurensin-2 (Fig. 3d). In contrast, the level of its isoform, Neurensin-1, was not altered in these mice, supporting the idea that the repression of Neurensin-2 by SMARCA3 in these cells is specific (Fig. S3c). These results confirm that in PV cells SMARCA3 selectively represses the expression of Neurensin-2. Furthermore, the downregulation of Neurensin-2 levels in response to chronic fluoxetine was completely abolished in SMARCA3 cKO mice (Fig. 3e), suggesting that SMARCA3 in PV cells mediates the reduction in Neurensin-2 level following chronic antidepressant treatment.

To test a possible role for SMARCA3 in PV cells in mediating the behavioral response to chronic SSRIs, we treated WT and SMARCA3 cKO mice with vehicle or fluoxetine for 21 days and tested their behaviors. WT mice, but not cKO mice, showed reduced immobility both in the forced swim test (FST), as well as in the tail suspension test (TST) after the SSRI treatment (Fig. 3f, g). cKO mice also failed to show fluoxetine-mediated reduced latency to bite a food pellet in the novelty suppressed feeding test (NSF, Fig. 3h). However, depressive-related behaviors and distance traveled in the open field were unchanged in cKO mice, ruling out the possibility that impairments in emotional or locomotor behaviors caused the deficit in the behavioral response to the SSRI (Figs. 3f–h,  S3d). Deletion of SMARCA3 from CCK cells also resulted in impaired response to chronic fluoxetine in the FST and NSF (Fig. S3e, f). In contrast to the loss of response to chronic treatment, the behavioral response to acute fluoxetine was unaffected when SMARCA3 was deleted either from PV or CCK cells (data not shown). Together, these data suggest that the p11/SMARCA3 pathway in PV interneurons mediates the response to chronic antidepressants.

### Repression of Neurensin-2 in DG PV neurons mediates the behavioral responses to chronic SSRIs

**Fig. 4:**
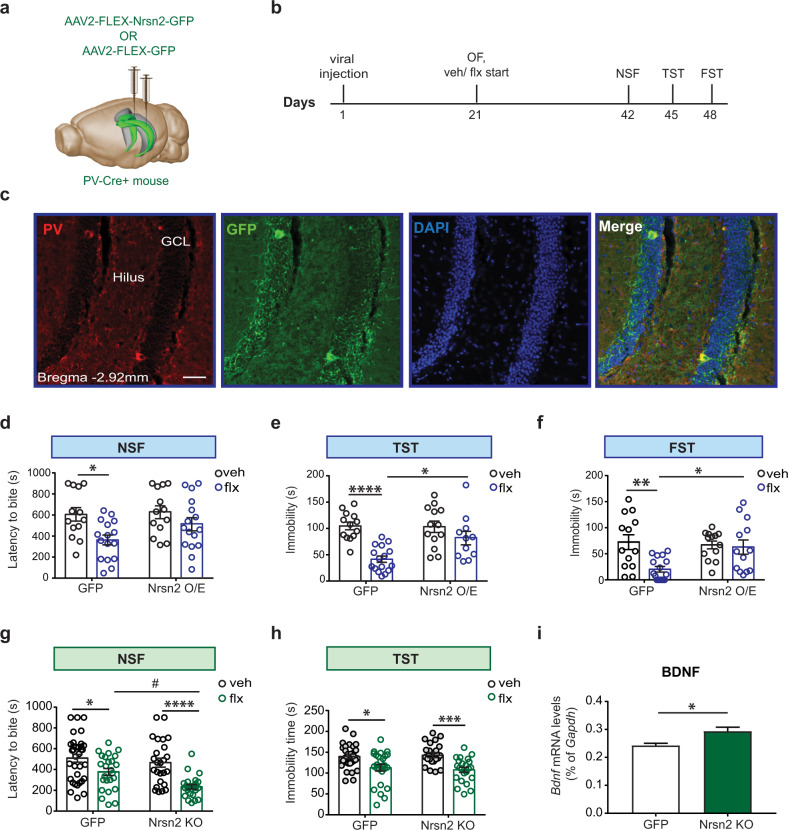
Neurensin-2 in PV cells mediates the behavioral response to SSRIs. **a** Schematic of the viral injection strategy. AAVs were injected bilaterally to the DG (in green) of PV-Cre mice. **b** Experimental design for viral expression of Neurensin-2, followed by chronic fluoxetine treatment and behavioral tests. **c** Representative images showing GFP expressing PV cells in the DG, 21 days after AAV2-FLEX-GFP injection. Scale bar, 50 μm. GCL granule cell layer. **d**–**f** Mice with viral-mediated overexpression of GFP or Neurensin-2 (Nrsn2 O/E) in DG PV cells were treated with vehicle (veh) or fluoxetine (flx) for 21 days (**d**) NSF. Two-way ANOVA; interaction *F* (1, 51) = 3.05, *P* = 0.087; virus, *F* = 4.4, *P* = 0.04; treatment, *F* = 6.18, *p* = 0.012. *p = 0.025, *n* = 13, 13, 16, 13. **e** TST. Two-way ANOVA; interaction, *F* (1, 49) = 5.19, *P* = 0.027; virus, *F* = 4.41, *P* = 0.040; treatment, *F* = 21.4, *P* < 0.0001. *p = 0.016, ****p < 0.0001. *n* = 13, 13, 16, 11. **f** FST. Two-way ANOVA; Interaction *F* (1, 51) = 5.31, *P* = 0.025; virus, *F* = 3.16, *P* = 0.08; treatment, *F* = 7.24, *P* = 0.009. **p = 0.004, *p = 0.023, *n* = 13, 13, 16, 13. **g**, **h** WT mice or mice with a constitutive deletion of Neurensin-2 (Nrsn2-KO) were treated for 21 days with vehicle (veh) or fluoxetine (flx) and tested behaviorally. **g** NSF. Two-way ANOVA; interaction, *F* (1, 104) = 1.99, *P* = 0.16; genotype, *F* (1, 104) = 6.62, *P* = 0.011; treatment, *F* (1, 104) = 25.8, *P* < 0.0001. *p = 0.039, #*p* = 0.038, ****p < 0.0001, *n* = 34, 25, 25, 24. **h** TST. Two-way ANOVA; interaction, *F* (1, 100) = 0.39, *P* = 0.53; genotype, *F* = 0.021, *P* = 0.88; treatment, *F* = 22.1, *P* < 0.0001. *p = 0.018, ***p = 0.0002, *n* = 31, 25, 25, 23. **i** Bar graph showing qPCR analysis of hippocampal *Bdnf* mRNA from WT and Nrsn2-KO mice. Unpaired *t* test, *p = 0.017 for Bdnf, *n* = 5–6/group.

We then studied whether Neurensin-2 in PV cells could be directly involved in the behavioral response to SSRIs. We used a viral strategy to overexpress Neurensin-2 in DG PV cells, and validated the upregulation of Neurensin-2 in these cells (Figs. 4a–c,  S4a). Then we treated these mice and GFP-transfected controls with chronic fluoxetine or vehicle, after which they were subjected to a set of behavioral tests (Fig. 4b). Strikingly, GFP induction, but not Neurensin-2 overexpression resulted in reduced latency to approach the food pellet in the NSF (Fig. 4d). In the TST and FST, mice that were transfected with the control GFP virus showed reduced immobility after the chronic treatment. Overexpression of Neurensin-2 completely prevented the reduced immobility by fluoxetine in both tests (Fig. 4e, f). Notably, overexpression of Neurensin-2 in PV cells did not affect basal despair-like behavior (Fig. 4e, f), feeding behavior (Fig. S4b), or locomotion (Fig. S4c). We then tested whether induction of Neurensin-2 in CCK cells might also compromise the behavioral response to chronic SSRI treatment. Viral-mediated overexpression of Neurensin-2 in DG CCK cells did not alter the behavioral response to chronic fluoxetine in both the TST and NSF (Fig. S4d–f). Taken together, these data support the idea that the downregulation of Neurensin-2 in DG PV cells, but not in CCK cells, is necessary for the behavioral response to SSRIs.

To examine whether downregulation of Neurensin-2 could alter the behavioral response to SSRIs, Neurensin-2 KO mice and WT controls were behaviorally tested in the NSF and TST tests after chronic treatment with fluoxetine. Neurensin-2 KO mice showed an augmented behavioral response to the SSRI in the NSF test, relative to WT mice (Fig. 4g). In the TST, the reduction in immobility was highly significant in KO mice (Fig. 4h), with no locomotor alternations (Fig. S5a).

Chronic antidepressant treatment is strongly associated with changes in DG circuitry [3]. To understand the role of Neurensin-2 in DG function, we measured the level of brain-derived neurotropic factor (BDNF), a known mediator of the response to antidepressants in the hippocampus [17]. The transcript levels of *Bdnf* in the hippocampus were induced by 19 ± 6% in hippocampi from KO mice, relative to WT mice (Fig. 4i), supporting the idea that suppression of Neurensin-2 level in the hippocampus could potentiate the response to antidepressants. Notably, *Bdnf* levels were upregulated following chronic fluoxetine in WT and KO mice (Fig. S5b).

### Chronic SSRIs increase AMPAR signaling in DG PV cells

**Fig. 5:**
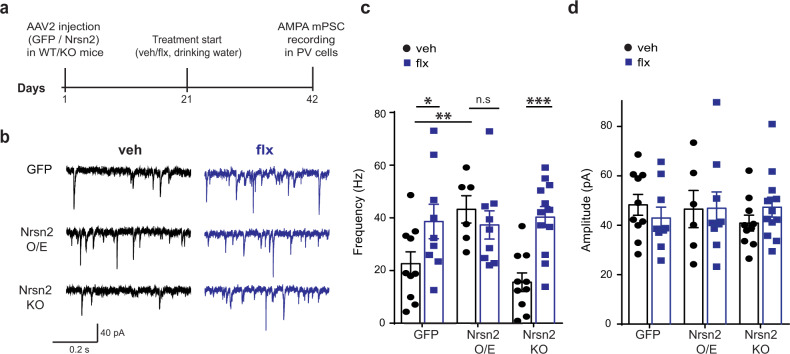
AMPAR signaling in PV cells is induced by chronic antidepressants and Neurensin-2. **a** Experimental design for viral expression of GFP or Neurensin-2 (Nrsn2 O/E), followed by chronic fluoxetine treatment and electrophysiological recordings. **b** Representative traces of AMPA mPSCs in DG SGZ PV neurons in mice with viral-mediated overexpression of GFP or Nrsn2 O/E in these cells, after 21 days of veh or flx treatment. Neurensin-2 KO (Nrsn2 KO) mice were injected with GFP to label cells. **c** Frequency of AMPA mPSCs in SGZ PV neurons. GFP-veh, *n* = 10 cells, 6 mice; GFP-flx, *n* = 9, 4; Nrsn2 O/E-veh, *n* = 6, 4; Nrsn2 O/E-flx, *n* = 9, 4, Nrsn2 KO-veh, *n* = 10, 5; Nrsn2 KO-flx, *n* = 12, 5. Two-way ANOVA followed by Fisher’s LSD post test, *F* (2, 50) = 4.71, Interaction, *P* = 0.01; treatment, *F* = 8.12, *P* = 0.006; virus, *F* = 3.13, *P* = 0.05. *p = 0.023, **p = 0.009, and ***p = 0.0003 for fluoxetine effect. **d** Amplitude of AMPA mPSCs in SGZ PV neurons. GFP-veh, *n* = 7 cells, 4 mice; GFP-flx, *n* = 9, 4; Nrsn2 O/E-veh, *n* = 6, 4; Nrsn2 O/E-flx, *n* = 9, 4; Nrsn2 KO-veh, *n* = 10, 5; Nrsn2 KO-flx, *n* = 12, 5. Two-way ANOVA followed by Fisher’s LSD post test, *F* (2, 50) = 0.81, interaction, *P* = 0.44; treatment, *F* = 0.01, *P* = 0.90; virus, *F* = 0.14, *P* = 0.86.

We recently reported a functional role for SMARCA3/Neurensin-2 pathway in regulating glutamate signaling in hippocampal interneurons. Deletion of SMARCA3 in CCK cells markedly attenuated the α-amino-3-hydroxy-5-methyl-4-isoxazolepropionic acid receptor (AMPAR) signaling in these cells [25]. To test whether SMARCA3 regulates a similar function in PV cells, we recorded AMPAR-mediated mPSCs in PV cells from WT and cKO mice. The deletion of SMARCA3 in PV cells resulted in a dramatic reduction of 59% and 12% in the respective frequency and amplitude of AMPA-mediated mPSCs in these interneurons (Fig. S6a–c), indicating that in PV cells, SMARCA3 is an important regulator of AMPAR signaling. Finally, we examined a possible role for the SSRIs-induced p11/SMARCA3/Neurensin-2 pathway activation in regulating the function of DG PV cells. To this end, we examined the effect of chronic treatment with fluoxetine on AMPAR signaling in these cells. We overexpressed GFP or Neurensin-2 in DG PV cells of WT mice, or expressed GFP in PV cells of KO mice, and treated these mice with vehicle or fluoxetine for 21 days, after which we recorded AMPAR-mediated mPSCs in the visibly transfected PV cells (Fig. 5a). Importantly, in WT mice, chronic fluoxetine resulted in a 72% increase in the frequency of AMPAR-mPSCs (Fig. 5b, c). Moreover, a dramatic increase of 160% in the frequency of the AMPAR-mediated currents was observed in PV cells from Neurensin-2 KO mice following the antidepressant. In sharp contrast, the fluoxetine-mediated increase in mPSC frequency was lost in PV cells following overexpression of Neurensin-2 (Fig. 5b, c). Notably, the frequency of the AMPAR-mediated currents was increased in PV neurons overexpressing Neurensin-2 compared with that in those transfected with the control GFP and no significant changes in amplitudes were detected in these experiments (Fig. 5b–d). Taken together, these data support the ideas that chronic antidepressant treatment potentiates glutamatergic AMPAR signaling in DG PV cells, and that this effect is dependent on the repression of Neurensin-2 expression in these cells.

## Discussion

Here we identified a novel, dynamic, and cell-type-specific signaling pathway, which is activated by chronic SSRIs in the DG PV interneurons to regulate AMPAR signaling and behavior. We report that the repression of Neurensin-2 in DG PV cells is indispensable for the behavioral response to SSRIs and characterize the cell-type specific functional changes involved in this response.

### Opposing dynamics of SMARCA3 and Neurensin-2 by stress and SSRIs

Neurensin-2 was downregulated by chronic treatment with antidepressants of different classes, including serotonergic, tricyclic and tetracyclic antidepressants. This downregulation of Neurensin-2 levels was detected in the hippocampus and in the prefrontal cortex. Previous reports identified these two brain regions as critical for the behavioral response to antidepressants [27, 29, 30, 32]. Neurensin-2 expression was reduced after 7 days of treatment, which coincides with the upregulation in p11 levels in these brain regions [27, 29, 30], suggesting that p11 has a role in the repression of Neurensin-2 expression after chronic treatment with SSRIs. Indeed, when we overexpressed p11 in vitro, Nrsn2 transcription was downregulated. Moreover, the constitutive deletion of p11 resulted in increased expression levels of Neurensin-2 in the hippocampus, indicating that p11 regulates Neurensin-2 expression both in vivo and in vitro.

In a companion paper, we found that the binding partner of p11, SMARCA3, represses Neurensin-2 in neurons. Furthermore, the deletion of SMARCA3 completely prevented the SSRI-mediated reduction in Neurensin-2 levels. These data suggest that the transcription of Neurensin-2 is dynamically inhibited by the p11-SMARCA3 pathway following antidepressant treatment. Our findings are adherent with a recent analysis of gene expression changes in the dorsal and ventral DG following chronic fluoxetine, showing a 50% reduction in *Nrsn2* expression and 100% upregulation in p11 levels, with no change in SMARCA3 expression [33]. We previously identified p11 as a dynamic mood-mediator protein that is downregulated by stress but is upregulated by antidepressants in both animal models and in humans [20]. A recent study showed that the antidepressive effects of p11 after SSRIs depends on its association with SMARCA3 in the hippocampus [24]. Here we show that in the adult brain, the subcellular distribution of SMARCA3, but not its cellular-expression level, is dynamically regulated by chronic treatment with SSRI. Our study indicates that the level of Neurensin-2, a newly identified downstream target of SMARCA3, negatively correlates with the nuclear localization of SMARCA3 under both stress and fluoxetine treatment. These data suggest that the activity of SMARCA3 is dynamically regulated by its nuclear localization. This dynamic subcellular localization of SMARCA3 is supported by the previous report showing that in vitro, p11 enhances SMARCA3 localization to the nuclear matrix, and its DNA binding efficacy [24]. Dynamic activity of chromatin remodelers in response to chronic stress was previously reported in a different limbic brain region, the nucleus accumbens. A transcriptional upregulation of ACF chromatin remodeling complex subunit mediated gene repression activity, and led to depressive-like behavior following stress [34]. Nonetheless, the mechanisms that dynamically regulate the subcellular localization of chromatin remodelers are poorly characterized. hSNF5, a member of the SWI/SNF remodelers family is exported from the nucleus to regulate the cytosolic shuttling process in mammalian cell culture [35]. In yeast, reduced nuclear localization of Swi3 and Snf5/6/11/12 was reported following hypoxia and was restored following reoxygenation [36]. The translocation of SMARCA3 out of the nucleus was previously linked to human thyroid cancer progression, as cytosolic localization of SMARCA3 was associated with reduced DNA repair-potential [37]. Based on our results, we suggest that in neurons the cellular localization and activity of SMARCA3 is bidirectionally responsive to sustained changes in environmental conditions such as chronic stress and to chronic SSRIs. These changes are essential in mediating the therapeutic response to antidepressants.

### Changes in Neurensin-2 levels by stress and following antidepressant treatment occur in different cell types

The reduction in Neurensin-2 following chronic SSRIs was crucial to induce an antidepressive-like behavior. Moreover, we found that the reduction in Neurensin-2 levels in PV cells, but not in CCK cells, mediated this behavioral response. Therefore, we suggest that the p11/SMARCA3/Neurensin-2 pathway is activated by chronic SSRIs in PV cells. In line with this, mice with a deletion of p11 in CCK cells showed the expected induction in p11 levels following the chronic treatment. However, deletion of p11 from PV cells prevented this molecular response. This indicates that in the hippocampus, SSRIs-induced upregulation in p11 levels occurs in PV cells. The deletion of SMARCA3 from either CCK cells or PV cells resulted in loss of behavioral response to chronic SSRI. These seemingly puzzling results could be explained by the robust depressive-like behavior seen in the mice with deletion of SMARCA3 from CCK cells [25]. This intrinsic depressive-like behavior may be resistant to antidepressants.

Overexpression of Neurensin-2 in DG PV cells resulted in lack of behavioral response to chronic fluoxetine, but their basal behavior was not depressive-like. On the contrary, in a companion paper, we reported notable depressive- and anxiety-like behaviors following the upregulation of Neurensin-2 in DG CCK cells, and here we confirmed that these mice retain normal behavioral response to the SSRI [25]. Collectively, the expression of Neurensin-2 in CCK and PV interneurons is highly dynamic and is regulated by similar molecular mechanisms. Nonetheless, chronic antidepressants downregulate Neurensin-2 in PV cells, whereas chronic stress upregulates its levels in CCK cells. How could a similar molecular pathway be responsive to different environmental changes in a cell-type specific manner? The different properties of these two interneuron populations might underlay their different roles in behavior. CCK interneurons are derived from the caudal ganglionic eminence and highly express postsynaptic cholinergic, endocannabinoid and several subtypes of serotonergic receptors [30, 38], all are highly associated with emotional behavior [30, 39, 40], subjecting these cells to be constantly responsive to changes in their microenvironment. While endocannabinoid and some serotonergic receptors mediate inhibition of CCK cells that lead to antidepressive-like behaviors [30], the cholinergic receptors activate CCK cells [41], which results in modulation in both anxiety and depressive-like behaviors [39, 42]. Moreover, CCK cells in the DG are directly innervated by serotonergic projections arising from the Raphe nuclei and are responsible for the acute antidepressive-like effect of SSRIs [30]. As CCK cells converge transmissions from multiple modulatory systems, they integrate information which regularly affects their intracellular signaling and cell activity [38]. Therefore, these cells are well-positioned to regulate basal-state behaviors that respond to constant environmental changes, including depressive-like behaviors. PV cells, on the other hand, are driven from the medial ganglionic eminence, and do not express any of these receptors on their postsynaptic surface [38]. This property suggests that the activity of PV cells is less responsive to local neuromodulators. Hence, these cells are less likely to mediate environmentally-derived depressive-like behaviors. Nonetheless, molecular and functional adaptations in PV cells are implicated in the response to chronic, but not acute SSRIs [22]. Following chronic SSRIs, serotonergic receptors emerge on PV cell surface and mediate the attenuated activity of these cells by serotonin [22]. Therefore, the different intrinsic pharmacological and connectivity properties of these two cell types likely underlie their involvement in distinct regulation of behaviors.

### Antidepressants and Neurensin-2 modulate AMPAR signaling in hippocampal interneurons

Chronic fluoxetine treatment resulted in increased AMPAR signaling in DG PV neurons. This finding may have an important implication in light of the fact that depression is highly associated with impairments in glutamatergic signaling [32], suggesting that its most effective treatment might alleviate this deficit. Moreover, we found that the deletion of SMARCA3 both in PV cells and in CCK cells (in the companion paper), resulted in impairments in AMPAR signaling, supporting the idea that AMPAR signaling is a major cellular function regulated by the p11/SMARCA3 pathway in these cells. The induction in the frequency of AMPAR-mediated currents following chronic SSRIs abolished when Nuerensin-2 was overexpressed in these cells, but was augmented when Neurensin-2 was deleted. Surprisingly, although Neurensin-2 was repressed after chronic fluoxetine, its overexpression in DG PV cells induced the frequency of AMPAR-mPSCs. A similar effect by Neurensin-2 overexpression was found in DG CCK cells (companion paper), indicating that Neurensin-2 is a regulator of AMPAR signaling in inhibitory interneurons. The exact mechanisms that underlie these discrepancies will require further studies. The massive changes in the frequency of AMPAR-mediated mPSCs by both SSRIs and Neurensin-2 suggest that both pre-and postsynaptic properties of the excitatory synapses in PV cells are dynamically modulated in the adult brain. The molecular mechanisms that regulate AMPAR signaling in DG PV cells of the adult brain are complex and largely unknown [43]. In the developing brain, AMPA receptors in PV cells regulate both pre- and postsynaptic properties of the excitatory synapse. AMPA receptors also influence the distribution of the glutamatergic synapses in somatic and dendritic locations, resulting in altered frequency of synaptic currents [44]. Here, for the first time, we show that chronic fluoxetine dramatically increases the frequency of AMPAR-mediated currents in these cells. This could be a result of multiple changes at different cellular and circuit levels [3], and, changes in spontaneous AMPAR signaling in PV neurons could possibly involve both pre- and postsynaptic adaptations in several cell types within and outside of the DG. For example, both newborn granule cells and mature granule cells are strongly associated with functional changes following antidepressant treatments [5, 7, 45]. In addition, accumulating recent data strongly supports the idea that DG interneurons undergo massive molecular and functional changes following chronic antidepressant treatments [22, 30, 31]. Therefore, future studies are needed in order to elucidate the exact molecular mechanisms behind the synaptic changes induced by Neurensin-2 and fluoxetine in PV cells. The precise molecular mechanism by which Neurensin-2 induces AMPAR transmission is yet to be elucidated. In a companion paper we showed that Neurensin-2 associates with the Homer proteins and the Arp2/3 complex in vitro. Homer proteins form scaffold complexes at the excitatory synapse, which promotes maturation of glutamatergic synapses, triggers the recruitment of synaptic AMPAR, and increases the synaptic strength [46, 47]. Moreover, it was previously shown that the endocytosis of AMPAR at postsynaptic sites is mediated by Homer proteins [46] and by the Arp2/3 complex [48]. The association of Neurensin-2 with these postsynaptic proteins and with endocytosis-related proteins suggests that it could regulate the endocytic cycle of AMPAR at the post synapse and hence, the induction of postsynaptic sites containing AMPAR in DG interneurons. Indeed, Neurensin-2 overexpression in PV interneurons altered AMPAR-mediated currents and prevented the fluoxetine-mediated induction of these changes. Future studies are indicated in order to elucidate the detailed mechanism by which Neurensin-2 regulates glutamatergic singling.

The cellular functions mediated by Neurensin-2 are poorly known. Its postnatal neuronal expression indicates that it might regulate synaptic functions [49]. Several studies in cancer models suggest that Neurensin-2 regulates the PI3K/AKT/mTOR pathway in an unknown mechanism [50–52]. In the context of depression, this pathway is specifically implicated in the induction of the synaptic translation of AMPAR and its incorporation into the postsynaptic membrane to form new synapses [13]. Importantly, this process was induced following treatment with a fast-acting antidepressant, and was mediated by BDNF [13, 53]. In the hippocampus, fast-acting antidepressant treatment was also linked to the activation of AMPAR [54]. Interestingly, Neurensin-2 KO mice showed induced levels of BDNF, suggesting that this pathway may be involved in the augmented behavioral response to SSRIs seen in the KO mice. Future studies are needed in order to understand if Neurensin-2 modulates the PI3K/AKT/mTOR pathway and BDNF signaling in hippocampal interneurons.

In conclusion, we identified a cell-type specific signaling pathway that mediates AMPAR signaling in PV interneurons and tightly modulates the action of antidepressants. Better understanding of the neuronal functions of Neurensin-2 could pave the way to the development of novel specific and effective diagnostics tools and antidepressant treatments.

## Supplementary information

Supplemental materials and methods

Supplemental figures
