# Supplementary material for: Activation of the p11/SMARCA3/Neurensin-2 pathway in parvalbumin interneurons mediates the response to chronic antidepressants
Source: Mol Psychiatry. 2021 Mar 15;26(7):3350–62. doi: 10.1038/s41380-021-01059-4 (PMC8505248; doi:10.1038/s41380-021-01059-4)
Supplement: Supplementary file 1 — Supplemental materials and methods [file 41380_2021_1059_MOESM1_ESM.docx]

**Supplemental Methods**

**Cell culture**

1x10^5^ N2a cells (ATCC, Manassas, VA, USA) were seeded in a 12-wells dish with cell media supplemented with 10% serum, 100 μg/mL Penicillin and 100 μg/mL Streptomycin and incubated at 37°C in a 5% CO_2_ air atmosphere. As cells reached 80% confluence, p11 or SMARCA3 plasmids (Genecopoeia, Rockville, MD, USA) were transfected using Lipofectamine 2000 (Thermo Fisher Scientific, Waltham, MA, USA). SMARCA3 siRNA was applied to induce knockdown (Silencer Select siRNAs, Thermo Fisher Scientific, Waltham, MA, USA). 36 h post transfection, cells were collected and lysed.

**TRAP quantitative PCR (qPCR)**

Translating Ribosome Affinity Purification (TRAP) was conducted as previously described ^1^. Briefly, 16 freshly harvested hippocampi from four PV^TRAP^ mice were pooled for one TRAP sample. RNA was purified using RNeasy Micro Kit (Qiagen, Hilden, Germany). All RNA samples were validated for high quality and were quantified using Bioanalyzer RNA 6000 Pico Kit (Agilent, San Diego, CA).

**Quantitative PCR (qPCR)**

For qPCR analysis, mRNA was extracted using commercial kit (Qiagen, Hilden, Germany­­­­­). cDNA was generated using High-Capacity cDNA Reverse Transcription Kit (Thermo Fisher Scientific, Waltham, MA, USA). 20ng of cDNA was used as a template for analysis. TaqMan Gene Expression Assays and Taqman Universal PCR Master Mix, no AmpErase UNG (Life Technologies, Carlsbad, CA) was used for all analyses. Fluorescence was detected using ABI 7900HT (Applied Biosystems, Foster City, CA). Changes in gene expression were calculated using the ∆∆ Ct formula.

**Behavioral assays**

Behavioral tests were performed during the light cycle in a designated sound-proof behavioral room by experimenters blinded to the genotype of animals and drug treatments. Adult (9-12 week old) male mice were used for all experiments. Stressed mice were tested in the following order: Social interaction (SI), Novelty suppressed feeding (NSF), sucrose preference test (SPT). Neurensin-2 KO mice were tested in the following order: Locomotor evaluation, NSF, TST. AAV injected mice and SMARCA3 cKO mice were tested in the following order: locomotor evaluation, NSF, TST, FST. At least 48 hours of interval were allowed between tests. All tested mice were habituated in the test room for at least 1 h prior to the test.

*Tail suspension test (TST)*

Immobility time was examined as previously described ^2^. Mice were suspended by the tail for 6 min and the immobility time was analyzed during the last 4 min by the automated TST analysis software (CleverSys Inc, Reston, VA, USA).

*Forced swim test (FST)*

Immobility in the FST time was examined as previously described ^3^ . Mice were placed in glass cylinder (16 cm diameter, 50 cm height) filled with water (23−24°C) to a height of 30 cm for 6 min. Immobility time was analyzed during the last 4 min by the automated FST analysis software (CleverSys Inc, Reston, VA, USA).

*Sucrose preference test (SPT)*

Singly housed mice were habituated to two water bottles for 24 h. The next day, 1 h before the dark period, one random water bottle was replaced with 1.5% sucrose solution. The consumption of water and sucrose solution was measured 12 h later by weighing the bottles. The sucrose preference was calculated as follows: (sucrose weight)/ (sucrose weight +water weight) x 100%.

*Locomotion*

Locomotor activity was examined as previously described ^2^. Mice were allowed to freely explore the open field arena (50 cm x 50 cm x 22.5 cm). Total distance traveled during a 60 min test session was automatically recorded and calculated using the automated Superflex software (Accuscan Instruments, Columbus, OH, USA).

*Novelty suppressed feeding (NSF)*

The latency to bite a food pellet under hunger conditions was measured as previously described ^4^. 24 h following food deprivation a single rodent-food pellet (2cm x 1cm) was placed in the center of an open field arena. The experimental mouse was placed in the arena and latency to first bite was recorded manually.

*Chronic social defeat stress (CSDS)*

The CSDS was carried out as previously described ^5^. For 10 consecutive days, the experimental mice were allowed to physically interact for 5 min with a previously screened, unfamiliar, aggressive CD-1 mouse in its home cage. Following this short physical stress, the two mice were separated by a perforated divider for the remaining 24 h until the next defeat session. Each day, the experimental mouse was exposed to a different novel aggressor. At the same time, control mice were placed in pairs within an identical setting of divided cages and were rotated daily between cages with no physical contact with one another. Fluoxetine or vehicle treatment started in a new home cage, a day after the social interaction test.

*Social interaction (SI)*

24 h after the last CSDS session, SI test was performed ^5^. SI test was composed of two phases, 2.5 min each, in which the experimental mice were allowed to explore an open field arena (42 cm x 42 cm x42 cm) with a wire mesh (10 cm wide x 6.5 cm deep x 42 cm high) located in a designated place inside the arena. In the first phase, the wire mesh was empty. In the second phase, a novel CD-1 aggressor mouse was placed inside the wire mesh. The amount of time spent by the experimental mice in the interaction zone (IZ) surrounding the wire mesh was recorded and analyzed by the video-tracking apparatus and software Ethnovision 7.0 (Noldus, Wageningen, the Netherlands). SI ratio was calculated by dividing the amount of time spent by the experimental mice in the IZ in the second phase over the time in the first phase. Susceptible mice were defined by a SI ratio < 1 whereas resilient mice were defined by a SI ratio >1.

*Restraint stress (RS)*

The RS was carried out as described previously, with mild changes ^6^. Briefly, mice were individually placed head first into perforated 50 mL polypropylene conical tubes, and then plugged with a 4.5-cm-long tube and tied with the cap of the 50 mL tube. The restraint stress episode lasted 3 h daily at the same time of the day, for 16 days. After each restrain session, mice were returned to their home cages. Control mice were housed in the same room without restraint.

**Immunohistochemistry**

Animals were deeply anesthetized and were slowly perfused transcardially with PBS, followed by 4% paraformaldehyde (PFA) in PBS. Brains were post-fixed in 4% PFA for 1 h at 4ºC, and then incubated overnight with 30% sucrose. Then, brains were frozen in the OCT medium over dry ice block and stored at -80ᵒC. Coronal sections of 20 μm were obtained using Leica CM3050 S cryostat and were frozen for later use. Sections were then thawed to room temperature and fixed with 4% PFA for 15 min, followed by 3 PBS washes and 1 hour block in 5% normal goat serum. Commercial primary antibodies against Neurensin-2 (mouse monoclonal, Sigma Aldrich, St. Louis, MO, 1:500), PV (Rabbit polyclonal, Abcam, Cambridge, MA, 1:1000) or GFP (Rabbit polyclonal, Thermo Fisher Scientific) were used for overnight incubation. For detection, secondary Alexa goat anti- mouse or goat anti-rabbit were used (Thermo Fisher Scientific). Finally, slices were incubated with DAPI (Roche, Mannheim, Germany, 1 μg/mL) for 10 min. Images were taken by a LSM710 confocal microscope (Zeiss, Germany).

**Western blotting**

Mice hippocampi were flash frozen and kept in -80ᵒC until use. For whole cell lysates, the hippocampi were added in 10 times excess volume of RIPA lysis buffer (Sigma Aldrich), supplemented with a protease inhibitor cocktail (Complete-EDTAfree; Roche), homogenized and sonicated with probe-type sonicator (Branson, Danbury, CT) for 10 sec twice, followed by centrifugation (5,000 rpm, 10 min). For cytosolic/nuclear fractionation, hippocampi were processed using NE-PER Nuclear and Cytoplasmic Extraction Reagents (Thermo Fisher Scientific). Protein level was determined by BCA protein assay (Thermo Fisher Scientific) and 20-30 μg protein were loaded onto 4–12% Tris-Glycine gels followed by a transfer to a 0.2 µm nitrocellulose membrane. For p11 detection, 50 µg of total protein was loaded on 16% Tricine gels and proteins were transferred to 0.2 µm PVDF. Proteins were detected using antibodies for Neurensin-2 (mouse monoclonal, Sigma Aldrich,1:750), β actin (Rabbit polyclonal, Cell Signaling Technology, Danvers, MA, 1:2,000), SMARCA3 (Rabbit polyclonal, Bethyl Laboratories, Montgomery, TX, 1:2,000), Lamin B2 (mouse monoclonal, Santa Cruz, Dallas, TX, 1:1,000), Neurensin-1 (Elabscience Biotechnology, Houston, Texas, 1:1,000), and p11 (goat polyclonal, 1:200, R&D systems, Minneapolis, MN).

**AAV preparation and stereotaxic delivery**

A sequence of the mouse Nrsn2 gene flanked by an internal ribosome entry site (IRES) and GFP was inserted into pAAV.Flex plasmid and was validated by sequencing. rAAV2 Flex.Nrsn2-IRES GFP or rAAV2.Flex.GFP were packaged at the Virus Vector Core Facility, UNC (Chapel Hill, NC, USA). 8 week-old mice were injected with 1 μl of AAV to the DG. The needle was left in the injection site for five minutes for complete diffusion. Injection coordinates were: ±2.00, −2.92 and −2.20 mm lateral, posterior and ventral relative to Bregma, according to the Franklin and Paxinos Mouse Brain Atlas, 3rd edition. These coordinates resulted in viral-mediated expression across a wide-range of the DG, between Bregma-2.18 to Bregma -3.80. Three weeks post injection, behavioral tests or physiological recordings were conducted. Successful injections were verified by visualization of GFP under florescence microscope. For validation of transfections accuracy, GFP positive cells at the SGZ were counted.

**Hippocampal slice preparation and electrophysiology**

Mice 12-16 weeks of age were euthanized with CO_2_. After decapitation and removal of the brains, transversal slices (400 μm thickness) were cut using a Vibratome 1000 Plus (Leica Microsystems, USA) at 2 °C in a cutting solution containing (all in mM): 93 NMDG, 1.2 NaHPO_4_, 2.5 KCl, 0.5 CaCl_2_, 10 MgCl_2_, 30 NaHCO3, 20 HEPES, 25 glucose, 5 sodium ascorbate, 2 thiourea, and 3 sodium pyruvate, and saturated with 95% O_2_ and 5% CO_2_. After cutting, slices were left to recover for 15 min at 36°C and then for 2 -4 h at room temperature (RT) in the recording solution (see below). The extracellular solution used for recordings contained (all in mM): 125 NaCl, 25 NaHCO_3_, 2.5 KCl, 1.25 NaH2PO_4_, 2 CaCl_2_, 1 MgCl_2_ and 10 glucose (bubbled with 95% O_2_ and 5% CO_2_). The slice was placed in a recording chamber (RC-27L, Warner Instruments, USA) and constantly perfused with oxygenated aCSF at 24°C (TC-324B, Warner Instruments, USA) at a rate of 1.5–2.0 ml/min. GFP-positive PV cells in the SGZ were selected for recording based on fluorescent expression from genetic background or from viral injection, using an upright Olympus BX51WI microscope equipped with the appropriate filters (Olympus, Japan) and a SPECTRA X LED light engine (Lumencor, OR, USA). Whole-cell patch-clamp recordings were performed with a Multiclamp 700B/Digidata1550A system (Molecular Devices, Sunnyvale CA, USA) and with glass pipettes (King Precision Glass, Inc, Glass type 8250) pulled in a horizontal pipette puller (Narishige) to a resistance of 3–4 MΩ. The intracellular solution contained (all in mM): 126 K-gluconate, 4 NaCl, 1 MgSO_4_, 0.02 CaCl_2_, 0.1 BAPTA, 15 glucose, 5 HEPES, 3 ATP, 0.1 GTP (pH 7.3). Recordings of miniature excitatory postsynaptic AMPA currents (mAMPA PSCs) were performed in the presence of tetradotoxin (0.5 µM) to block Na+ currents, bicuculline (30 µM) to block GABA activity, and of APV (50 µM) to block NMDA-mediated excitatory currents. cKO-PV data are shown as pooled events from each cell per treatment, and viral injection data were averaged from top 25% of events per cell for normalization.

**Statistical analysis**

Unless otherwise indicated, statistical analysis for mice treated with fluoxetine or vehicle was conducted by two-way ANOVA followed by Tukey’s post hoc test. For one-way ANOVA, significance was determined by Bonferroni’s post hoc test. For slice recording in cKO-PV experiment, statistical analysis of was performed using Kolmogorov–Smirnov test. For slice recording in fluoxetine experiment, Two-way ANOVA followed by Fisher’s LSD post-test was applied. In all experiments, p < 0.05 was considered significant and the variance was similar between the groups that were statistically compared. Sample size was chosen based on previous reports to ensure adequate power and mice were randomly allocated to experimental groups. Statistical analysis was performed using GraphPad Prism 7.02. All data are represented as means ± SEM.

1. Sagi Y, Medrihan L, George K, Barney M, McCabe KA, Greengard P. Emergence of 5-HT5A signaling in parvalbumin neurons mediates delayed antidepressant action. *Mol Psychiatry* 2019.

2. Svenningsson P, Chergui K, Rachleff I, Flajolet M, Zhang X, El Yacoubi M *et al.* Alterations in 5-HT1B receptor function by p11 in depression-like states. *Science* 2006; **311**(5757)**:** 77-80.

3. Warner-Schmidt JL, Schmidt EF, Marshall JJ, Rubin AJ, Arango-Lievano M, Kaplitt MG *et al.* Cholinergic interneurons in the nucleus accumbens regulate depression-like behavior. *Proc Natl Acad Sci U S A* 2012; **109**(28)**:** 11360-11365.

4. Virk MS, Sagi Y, Medrihan L, Leung J, Kaplitt MG, Greengard P. Opposing roles for serotonin in cholinergic neurons of the ventral and dorsal striatum. *Proc Natl Acad Sci U S A* 2016; **113**(3)**:** 734-739.

5. Golden SA, Covington HE, Berton O, Russo SJ. A standardized protocol for repeated social defeat stress in mice. *Nat Protoc* 2011; **6**(8)**:** 1183-1191.

6. Oh YS, Gao P, Lee KW, Ceglia I, Seo JS, Zhang X *et al.* SMARCA3, a Chromatin-Remodeling Factor, Is Required for p11-Dependent Antidepressant Action. *Cell* 2013; **152**(4)**:** 831-843.
