# Supplementary material for: Activation of the p11/SMARCA3/Neurensin-2 pathway in parvalbumin interneurons mediates the response to chronic antidepressants
Source: Mol Psychiatry. 2021 Mar 15;26(7):3350–62. doi: 10.1038/s41380-021-01059-4 (PMC8505248; doi:10.1038/s41380-021-01059-4)
Supplement: Supplementary file 2 — Supplemental figures [file 41380_2021_1059_MOESM2_ESM.pdf]

Figure S1

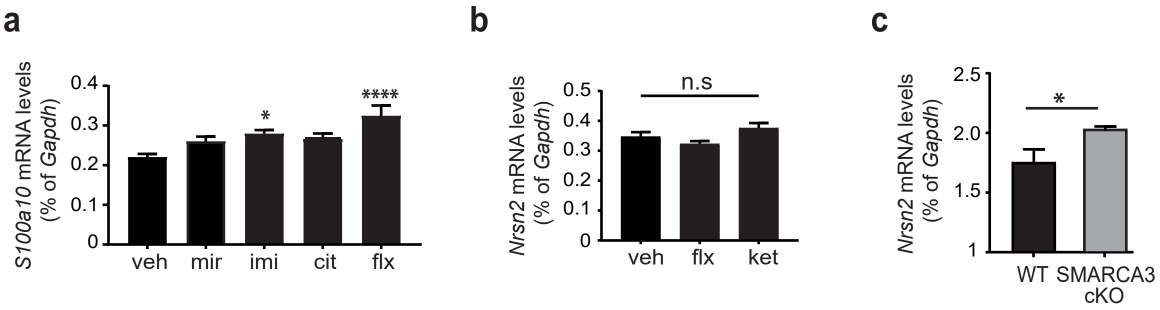
a
S100a10 mRNA levels (% of Gapdh)
0.4
0.3
0.2
0.1
0
*
****
veh
mir
imi
cit
flx
b
Nrsn2 mRNA levels (% of Gapdh)
n.s
0.5
0.4
0.3
0.2
0.1
0
veh
flx
ket
c
Nrsn2 mRNA levels (% of Gapdh)
2.5
2.0
1.5
1
*
WT
SMARCA3 cKO

Figure S2

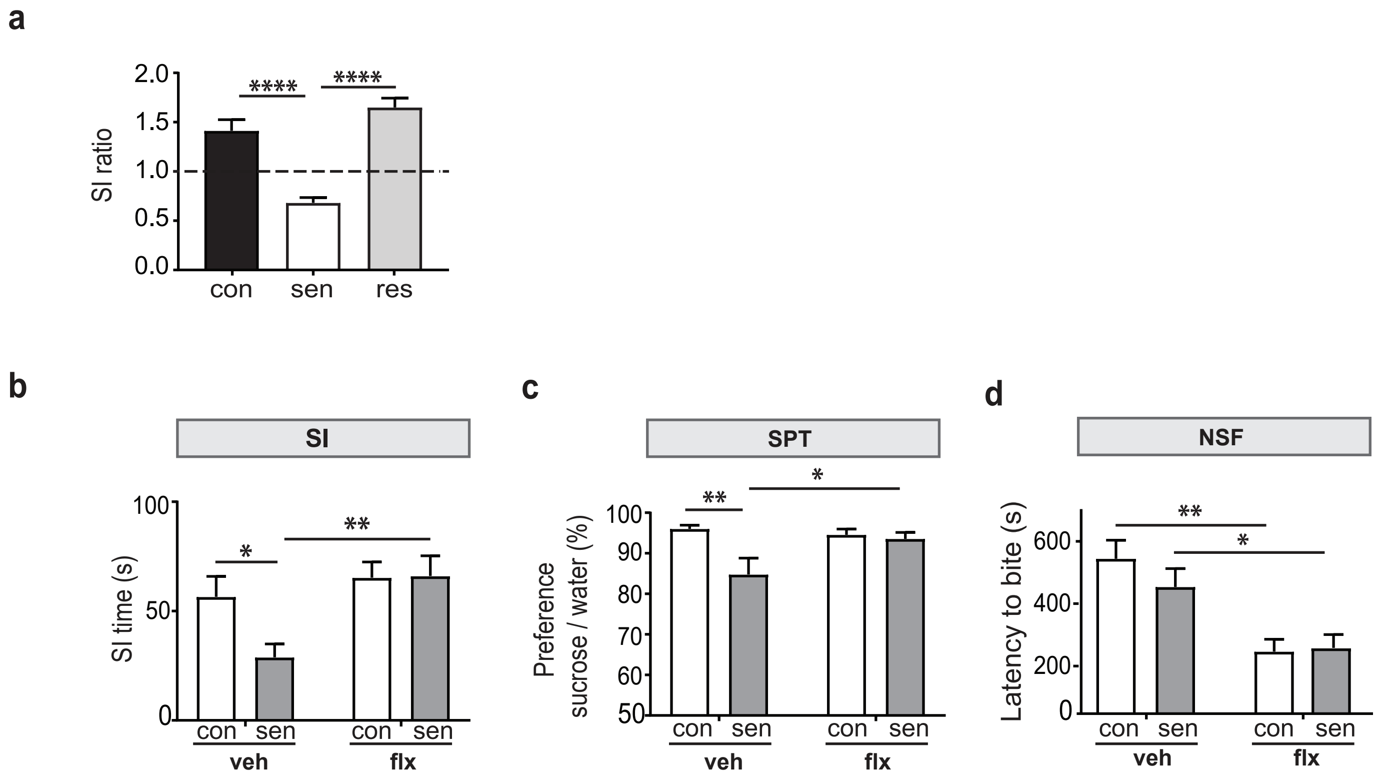
a
SI ratio
2.0
1.5
1.0
0.5
0.0
****
****
con
sen
res
b
SI
SI time (s)
100
50
0
*
**
con
sen
con
sen
veh
flx
c
SPT
Preference
sucrose / water (%)
100
90
80
70
60
50
**
*
con
sen
con
sen
veh
flx
d
NSF
Latency to bite (s)
600
400
200
0
**
*
con
sen
con
sen
veh
flx

Figure S3

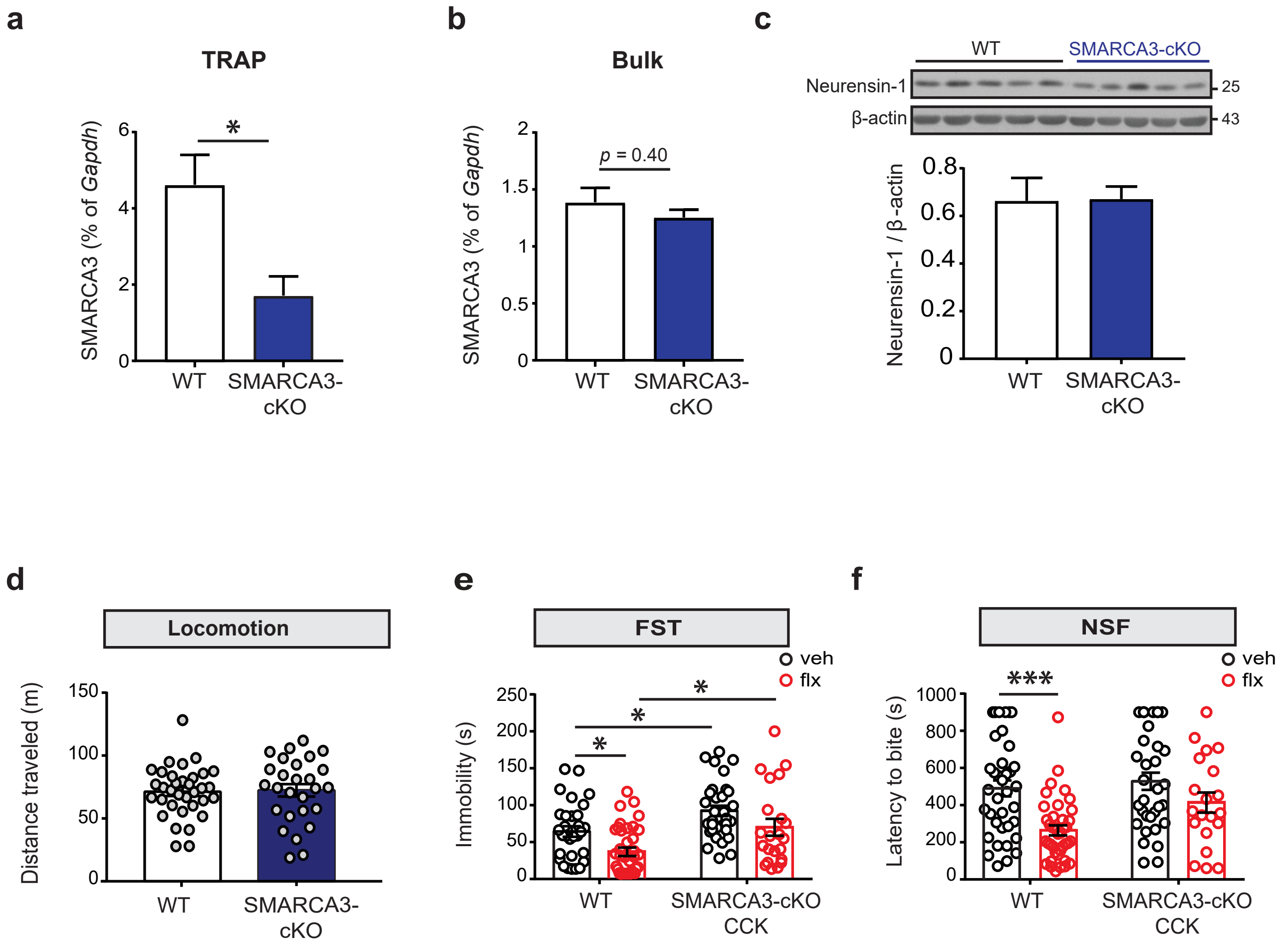

a
TRAP
SMARCA3 (% of Gapdh)
6
4
2
0
*
WT
SMARCA3-cKO
b
Bulk
SMARCA3 (% of Gapdh)
2
1.5
1
0.5
0
p = 0.40
WT
SMARCA3-cKO
c
WT
SMARCA3-cKO
Neurensin-1
β-actin
25
43
Neurensin-1 / β-actin
0.8
0.6
0.4
0.2
0
WT
SMARCA3-cKO
d
Locomotion
Distance traveled (m)
150
100
50
0
WT
SMARCA3-cKO
e
FST
veh
flx
Immobility (s)
250
200
150
100
50
0
*
*
*
WT
SMARCA3-cKO CCK
f
NSF
veh
flx
Latency to bite (s)
1000
800
600
400
200
0
***
WT
SMARCA3-cKO CCK

Figure S4

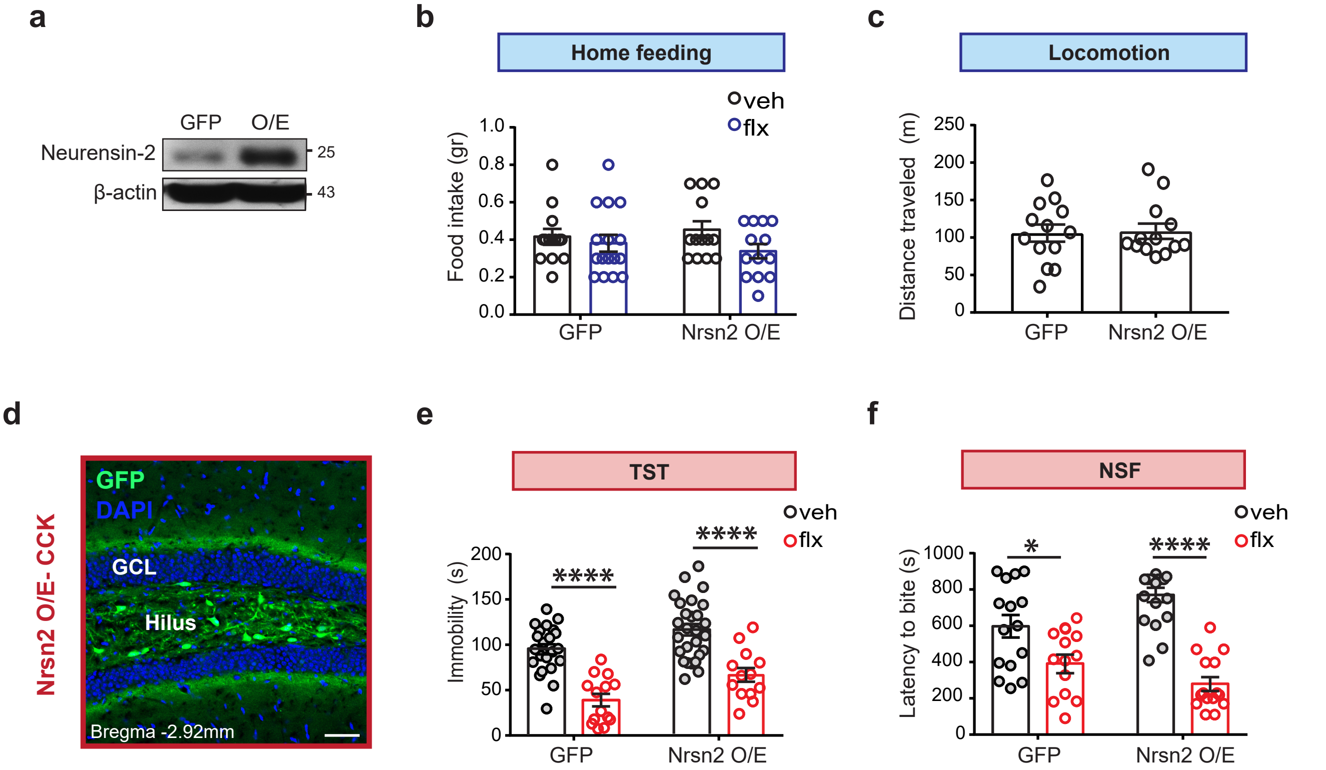

a
GFP
O/E
Neurensin-2
β-actin
25
43
b
Home feeding
veh
flx
Food intake (gr)
1.0
0.8
0.6
0.4
0.2
0.0
GFP
Nrsn2 O/E
c
Locomotion
Distance traveled (m)
250
200
150
100
50
0
GFP
Nrsn2 O/E
d
Nrsn2 O/E- CCK
GFP
DAPI
GCL
Hilus
Bregma -2.92mm
e
TST
veh
flx
Immobility (s)
200
150
100
50
0
****
****
GFP
Nrsn2 O/E
f
NSF
veh
flx
Latency to bite (s)
1000
800
600
400
200
0
*
****
GFP
Nrsn2 O/E

Figure S5

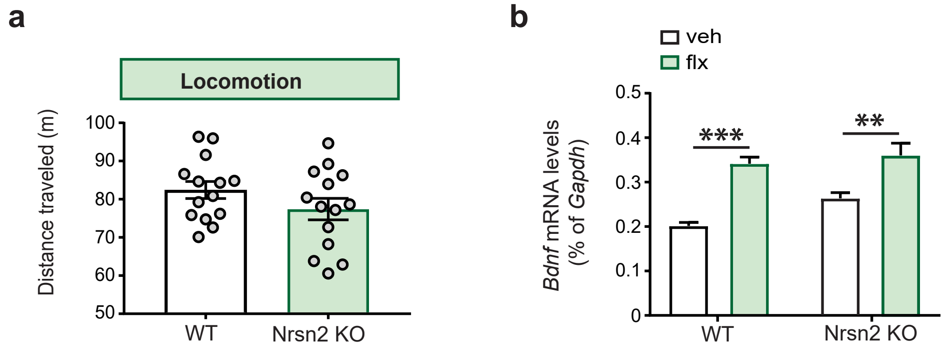
a
Locomotion
Distance traveled (m)
100
90
80
70
60
50
WT
Nrsn2 KO
b
veh
flx
Bdnf mRNA levels (% of *Gapdh*)
0.5
0.4
0.3
0.2
0.1
0
***
**
WT
Nrsn2 KO

Figure S6

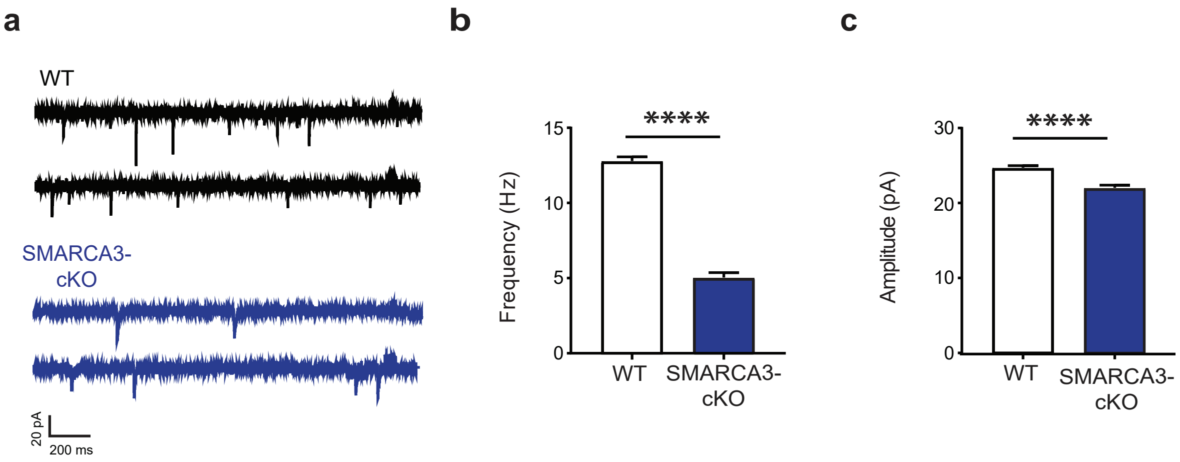
a
WT
SMARCA3-
cKO
20 pA
200 ms
b
****
15
10
5
0
Frequency (Hz)
WT
SMARCA3-
cKO
c
****
30
20
10
0
Amplitude (pA)
WT
SMARCA3-
cKO

Supplemental figure legends

Figure S1. Nrsn2 level is unchanged after acute antidepressant treatments. Related to Fig. 1

(a) Bar graph of qPCR analysis of p11 mRNA expression (*S100a10*) in the hippocampus after 14 days of daily intraperitoneal injection of vehicle (veh), mirtazapine (mir), imipramine (imi), citalopram (cit) and fluoxetine (flx). One-way ANOVA, F (4, 20) = 7.99, P = 0.0005; *p = 0.017 ****p = 0.0001. n = 5/group.

(b) Bar graph of qPCR analysis of Nrsn2 mRNA expression in the hippocampus 30 minutes after intraperitoneal injection of vehicle (veh), fluoxetine (flx) and ketamine (ket). One-way ANOVA, F (2, 12) = 3.85, P = 0.051; p = 0.36 for flx, p = 0.26 for ket. n = 5/group.

(c) Bar graph summarizing qPCR analysis of Nrsn2 mRNA expression in the hippocampus. RNA was extracted from hippocampi of WT and mice with SMARCA3 deletion in the nervous system, using Nestin-Cre mice (SMARCA3 cKO). Unpaired t-test, *p = 0.038. n = 5/group.

Figure S2. Fluoxetine reverses behavioral deficits in stress-sensitive mice. Related to Fig. 2

(a) Bar graph showing the interaction ratio in stress-naïve control (con) mice, or in stress-sensitive (sen) and resilient (res) mice in the social interaction test (SI) following chronic social defeat stress. One-way ANOVA; F (2, 58) = 30.74; ****p < 0.0001. n = 17-25/group.

(b-d) Stress naïve mice (con) and stress-sensitive (sen) mice after CSDS were treated with oral vehicle or fluoxetine for 21 days and tested behaviorally. n = 7-9/ group. (b) Social interaction (SI) test. Two-way ANOVA; interaction, F (1, 30) = 4.92, P = 0.034; stress, F = 4.49, P = 0.043; treatment, F = 6.87, P = 0.013. *p = 0.022, **p = 0.009. (c) Sucrose preference test (SPT). Two-way ANOVA; interaction, F (1, 29) = 4.94, P = 0.032; stress, F = 5.85, P = 0.022; treatment, F = 2.60, P = 0.117. **p = 0.009, *p = 0.045. (d) Novelty suppressed feeding (NSF). Two-way ANOVA; interaction, F (1, 30) = 0.94, P = 0.33; stress, F = 0.56, P = 0.45; treatment, F = 22.1, P < 0.0001. **p = 0.0025, *p = 0.048. n = 9/group.

Figure S3. SMARCA3 in PV cells mediates AMPAR signaling. Related to Fig. 3

(a-b) qPCR analysis of SMARCA3 transcript levels in PV cells (a), and in bulk hippocampal mRNA extraction (b). WT and cKO mice were subjected to TRAP and mRNA was isolated from PV hippocampal cells and from the unbound fraction (bulk). Unpaired t-test; * $p = 0.035$. $n = 3$ from 12 mice/group.

(c) Western blot scan (top) and quantification (bottom) of hippocampal Neurensin-1 protein expression in WT mice and in mice with SMARCA3 deletion in PV cells (cKO). t-test, $p = 0.94$, n= 5/group. Molecular weights are indicated in KDa.

(d) Bar graph of distance traveled in an open-field arena during a 60 min test in WT or mice with SMARCA3 deletion in PV cells. Unpaired t-test, $p = 0.85$, n = 27-34/group.

(e-f) Behavioral tests in WT or in mice with deletion of SMARCA3 in CCK cells (SMARCA3-cKO CCK) after 21 days of treatment with vehicle (veh) or fluoxetine (flx). (e) FST, forced swim test. Two-way ANOVA; Interaction F (1, 116) = 0.12, P = 0.72; genotype, F = 16.09, P = 0.0001; treatment, F = 10.74, P = 0.0014. *p = 0.037 fluoxetine effect in WT. *p = 0.041 genotype effect in vehicle treated mice, *p = 0.017 genotype effect in fluoxetine treated mice, n = 30, 36, 32, 22. (f) NSF, Novelty suppressed feeding. Two-way ANOVA; Interaction F (1, 125) = 1.67, P = 0.19; genotype, F = 4.80, P = 0.030; treatment, F = 15.82, P = 0.0001. ***p = 0.0003. n = 37, 38, 31, 21.

Figure S4. Regulation of Neurensin-2 in CCK cells does not mediate the response to fluoxetine. Related to Fig. 4

(a) Western blot image showing the expression of Neurensin-2 in the hippocampus 21 days after AAV-GFP (GFP) or AAV-Nrsn2-IRES-GFP injection (O/E). Molecular weights are indicated in KDa.

(b) Food consumption in home-cage following the NSF test. 20 days post injection, mice with viral-mediated expression of GFP or Nrsn2 O/E in DG PV cells, were treated for 21 days with veh or flx. Two

way ANOVA; interaction, F (1, 51) = 0.88, p = 0.35; virus, F = 0.003, p = 0.96; treatment, F = 2.98, p = 0.93. n = 13, 13, 16, 13.

(c) Locomotion in mice with viral-mediated expression of GFP or Nrsn2 O/E in DG PV cells. Mice were subjected to an open field arena and the distance they traveled during a 60 min period was recorded. Unpaired t-test, p = 0.87, n=13, 13.

(d) Representative image showing GFP expressing CCK cells in the DG, 20 days after AAV2-FLEX-GFP injection to CCK^{Cre} mouse. Scale bar, 50 µm. GCL, granule cell layer.

(e-f) TST and NSF in mice with viral-mediated expression of GFP or Nrsn2 O/E in DG CCK cells. 20 days post injection, mice started 21 days of treatment with vehicle (veh) or fluoxetine (flx). (e) TST. Two-way ANOVA; interaction, F (1, 72) = 0.21, P = 0.64; virus, F = 12.56, P = 0.0007; treatment, F = 57.67, P < 0.0001. ****p < 0.0001, n = 22, 27, 14, 13. (f) NSF. Two-way ANOVA; interaction, F (1, 56) = 8.60, P = 0.005; virus, F = 0.40, P = 0.53; treatment, F = 52.18, P < 0.0001. *p = 0.024, ****p < 0.0001, n= 15, 17, 15, 13.

Figure S5: No alterations in SSRIs- induced Bdnf upregulation in Nrsn2 KO mice. Related to Fig. 4

(a) Bar graphs of distance traveled in an open-field arena during a 60 min test by WT and Neurensin-2 KO mice (Nrsn2 KO). Unpaired t-test, p = 0.17, n = 14/group.

(b) qPCR analysis of *Bdnf* transcript levels in hippocampal mRNA extraction after chronic treatment with vehicle (veh) and fluoxetine (flx, 20mg/Kg/day, 14 days, i.p. injections). Two-way ANOVA; Interaction, F (1, 15) = 4.29. P = 0.26; treatment, F = 40.54, p < 0.0001; genotype, F = 4.75, P = 0.045. **p = 0.0049, ***p = 0.001. n= 4-5/group.

Fig. S6: SMARCA3 regulates AMPAR-mediated currents in PV cells. Related to Fig. 5

(a) Representative traces of AMPA mPSCs, as measured in PV neurons in the SGZ of WT and cKO mice.

(b-c) AMPA mPSCs frequency (b) and amplitude (c) in SGZ PV neurons from WT (n= 3639 events, 11 cells, 5 mice) and cKO (n=982, 10, 4). Kolmogorov–Smirnov test, ****$p < 0.0001$.
